# Augmentation for rotator cuff repair – clinical use patterns and limited patient access: the American Shoulder and Elbow Surgeons bio-advocacy work group survey

**DOI:** 10.1016/j.xrrt.2026.100748

**Published:** 2026-04-10

**Authors:** Brandon D. Bushnell, Nathan Boes, Akin Cil, Kevin Farmer, Gregory Gilot, Hafiz Kassam, Adam Khan, Anthony Miniaci, Joshua Port, Joaquin Sanchez-Sotelo, Mark Schultzel, Scott Steinmann, Misty Suri, David Weinstein, Melissa Wright

**Affiliations:** a Atrium Harbin Clinic, Department of Orthopedic Surgery, Rome, GA, USA; b Apex Orthopedics, Frisco, TX, USA; c University of Missouri-Kansas City, Department of Orthopedic Surgery, Kansas City, MO, USA; d University of Florida, Department of Orthopedic Surgery, Gainesville, FL, USA; e Cleveland Clinic, Department of Orthopedic Surgery, Cleveland, OH, USA; f Hoag Orthopedic Institute, Newport Beach, CA, USA; g Kaiser Permanente Medical Group, Santa Monica, CA, USA; h Baptist Health Orthopedic Institute, Boca Raton, FL, USA; i University Orthopedics Center, Altoona, PA, USA; j Mayo Clinic, Department of Orthopedics, Rochester, MN, USA; k United Medical Doctors, San Diego, CA, USA; l Mayo Clinic, Department of Orthopedics (Emeritus), Rochester, MN, USA; m Oschner Andrews Sports Medicine Institute, Department of Orthopedic Surgery, New Orleans, LA, USA; n Colorado Center of Orthopedic Excellence, Colorado Springs, CO, USA; o Washington University St. Louis, Department of Orthopedic Surgery, St. Louis, MO, USA

**Keywords:** Rotator cuff repair, Augmentation, Bioinductive collagen implant, Human dermal allograft, Supplementation, Advocacy

## Abstract

**Background:**

Over the last decade, treatment algorithms of rotator cuff pathology have increasingly included various forms of augmentation of rotator cuff repair (RCR). This study aimed to quantify real-world clinical patterns for RCR augmentation and provide consensus statements for clinical practice and payor consideration. It was our hypothesis that augmentation would be popular amongst surgeons, especially for reduction in retear rates, and that a high percentage of respondents would also identify restrictions to access.

**Material and methods:**

The American Shoulder and Elbow Surgeons Advocacy Committee distributed a 12-question digital survey to all current members of American Shoulder and Elbow Surgeons. The survey evaluated current surgical techniques and augmentation usage, limitations on augmentation access, target patients for augmentation selection, and desired clinical outcomes. Questions were analyzed as either frequency of response or as a rank average with 95% confidence intervals.

**Results:**

The survey was sent to 1,210 surgeons, and 103 surgeons participated in the survey (8.5% response rate). The survey revealed the following: (1) use of RCR augmentation is reported by 76.2% and 85.1% of surgeons for partial-thickness tears (PTT) and full-thickness tears (FTT), respectively. However, 74.5% of surgeons indicate that they have limited or variable access to augmentation options. (2) A bioinductive collagen implant (BCI) is the most preferred form of augmentation for PTT (52.5% of respondents), while both the BCI (45.5%) and human dermal allograft augmentation (45%) are most preferred for FTT.(3) The decision to use augmentation is largely based on positive clinical outcomes (9.4/10) and a defined target patient population (8.4/10), with the most critical outcome being a lower retear rate for both PTT (7/10) and FTT (8/10). (4) For PTT, patient comorbidities (7/10) are of greatest concern and are the most impactful criteria for the decision to use augmentation (6/10). For FTT, poor tendon quality (8.6/10) and increasing tear size (2.9-9.1/10) are of greatest concern, with tear size indicated as the most impactful criteria for selecting augmentation (7.6/10).

**Conclusion:**

This expert-opinion survey confirmed the growing popularity of RCR augmentation and the significant limitations in access faced by surgeons and their patients. BCI and human dermal allograft were the most popular augmentation options. Surgeons identify multiple factors as important to decision-making for implant use, including positive clinical outcomes, low retear rates, defined patient populations, patient comorbidities, poor tendon quality, and tear size. Research in this area continues to expand, but additional work on payor approval remains to ensure appropriate access to this technology.

Nearly 5 million patients seek rotator cuff treatment in the United States each year, with over 1 million patients a year undergoing rotator cuff repair.^17^ Surgical repair of symptomatic tears has been identified as the most cost-effective management option over time,^25^ but retear rates and patient outcomes after surgical repair have remained relatively consistent in spite of significant proliferation of literature on repair techniques.^29^ Improving these outcomes and increasing longevity of the treatment is critical, as the economic burden of failed rotator cuff repairs is estimated at $440 million per year in the short term post-operative period.^45^

Over the last decade, augmentation of rotator cuff repairs has emerged as a potential solution to this problem. Numerous recent systematic reviews have documented the effectiveness of various biologic options in reducing retear rates and improving outcomes after rotator cuff repair.^13^^,^^28^^,^^43^ Recently, the American Academy of Orthopaedic Surgeons (AAOS) updated its Clinical Practice Guidelines (CPGs) to reflect the growing body of literature supporting biologic augmentation.^1^ Despite the incremental cost that biologics and implants can add to surgical procedures, the potential improvement in patient-reported outcomes and reduction in retear rates by using augmentation has led to a call for greater collaboration with payors to leverage these therapeutic advancements and make them more widely available to surgeons and patients.^28^^,^^32^

Even as evidence mounts in support of augmentation, real-world adoption of these technologies remains relatively unknown. Several factors can limit implementation of new technology, including poorly defined patient selection protocols, lack of supporting clinical evidence, or barriers to access, including cost or reimbursement. The American Shoulder and Elbow Surgeons (ASES) established a Bio-Advocacy Work Group with a stated mission to identify real-world practice patterns of rotator cuff augmentation and advocate on behalf of patients and surgeons to ensure that medical decision-making is driven by the best evidence supporting optimal clinical outcomes. In alignment with these objectives, the Work Group designed an expert-opinion survey study whose purpose was to assess common practices and preferences of surgeons for the augmentation of rotator cuff repairs (RCRs) and availability of this technology for patients. It was our hypothesis that augmentation would be popular among surgeons, that the most common indication would be a reduction in retear rates, and that a high percentage of respondents would also identify restrictions to access.

## Methods

The ASES Bio-Advocacy Work Group, consisting of 15 members, reviewed current literature and CPGs and created a digital survey to evaluate the following aspects of management for both partial-thickness tears (PTT) and full-thickness tears (FTT) of the rotator cuff: (1) current surgical intervention techniques; (2) preferred methods for augmentation; (3) patient selection criteria for using augmentation; (4) desired clinical benefits from biologic implant/device; and (5) limitations in access to biologic implant/device technologies (Appendix 1 – Survey Questions). The survey consisted of 12 questions with a variety of multiple-choice or ranking questions. The first 2 questions investigated whether surgeons use any options within a broad category of biologic augmentation methods, including platelet rich plasma (PRP), bioinductive collagen implants (BCI), subacromial balloon spacer devices, bone marrow aspirate concentrate, synthetic material patches, human dermal allograft (HDA) patches, and other methods. The remaining 10 questions focused on access to technology, preferred treatment methods, and factors that influence decision-making for use of augmentation, including available information about the option, patient characteristics, and key outcomes. The Work Group distributed the digital survey to all active ASES members in Spring 2025. ASES societal membership is a competitive process exclusive to orthopedic surgeons with a primary practice focus on shoulder and elbow, with demonstrated expertise in this subject area. Respondents had one opportunity to complete the survey and could not alter answers after submission. A total of 103 members in total responded to the survey, with varying numbers of respondents per individual question. Questions were analyzed as either frequency/percentage of response or as a rank average with a 95% confidence interval (CI). This study did not require institutional review board approval, as it did not directly involve patients or their records.

## Results

### Use of augmentation

**Figure 1:**
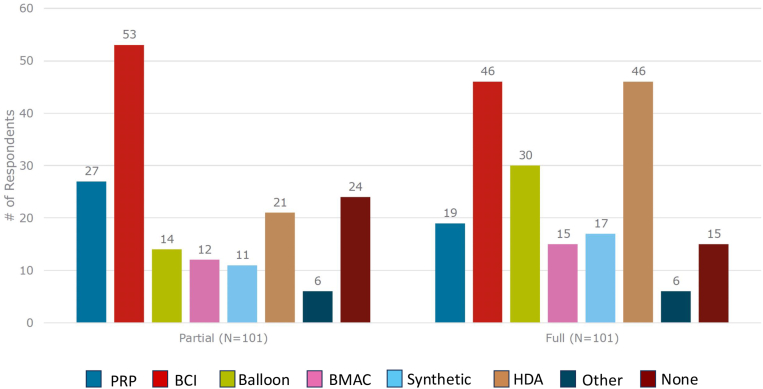
Surgeon utilization of biologic augmentation options for partial- and full-thickness rotator cuff tears. *PRP*, platelet rich plasma; *BCI*, bioinductive collagen implant; *balloon*, subacromial balloon spacer; *BMAC*, bone marrow aspirate concentrate; *synthetic*, synthetic material patch; *HDA*, human dermal allograft patch; *other*, additional biologic augmentation option not otherwise listed.

Most surgeons reported at least occasional use of some form of augmentation for RCRs for both PTT and FTT (Fig. 1). Augmentation is utilized by 76.2% of surgeons for PTT, with the most common methods of augmentation consisting of BCI (52.5%) and PRP (26.7%). Augmentation is utilized by 85.1% of surgeons for FTT, with the most common methods of augmentation consisting of BCI (45.5%) and HDA (45.5%).

### Access to augmentation options

Twenty-five percent of surgeons reported no clinical practice restrictions on the use of augmentation options. However, 74.5% of surgeons reported restrictions on access, with 35.6% reporting “limited” and 38.9% reporting “variable” access to augments based on surgical setting (eg, hospital vs. ambulatory surgery center [ASC]) (Table I). No surgeons completing the survey indicated a complete absence of access to augmentation technology.

**Table I:** Limitations on use of biologic augmentation.

Response	# of responses	Percentage
Which statement accurately describes your current selection and use of biologic devices/implants augmentation for the surgical treatment for rotator cuff tears based on insurance payment rates or methodologies (ie, packaged payments for all implants and supplies)?		
I have no restrictions or unaware of any restrictions on biologic devices/implants used for the surgical treatment of rotator cuff tears	23	25.6%
I am limited on the type of biologic devices/implants used for the surgical treatment of rotator cuff tears	32	35.6%
I have no access to biologic devices/implants used for the surgical treatment of rotator cuff tears	0	0.0%
My access to biologic devices/implants used for the surgical treatment of rotator cuff tears is variable based on setting of surgical care (ie, ASC vs. hospital)	35	38.9%

ASC, ambulatory surgery center.

### PTT

Surgical management for symptomatic PTT was preferred by 89.3% of surveyed surgeons, with 6% of surgeons electing to use only a biologic implant and 15.5% of surgeons electing to perform a sutured repair with biologic augmentation as their standard of care (Table II). When treating PTT, patient comorbidities present the greatest challenge to surgeons (mean: 7.0; 95% CI: 6.5-7.5). Presence of comorbidities was also the most impactful when determining to use augmentation (mean: 6.0; 95% CI: 5.4-6.7) as part of surgical management (Table III). Post-operatively, lower incidence of retear and/or revision procedures was the most critical outcome (mean: 7.0; 95% CI: 6.3-7.7) surgeons sought in their decision to use augmentation.

**Table II:** Management of PTT.

Response	# of responses	Percentage
What is your preferred method of surgical treatment for partial-thickness rotator cuff tears?		
This is not a surgical case; conservative treatment remains primary treatment option	9	10.7%
Subacromial decompression and débridement only	3	3.6%
No rotator cuff repair and isolated use of biologic device/implants	5	6.0%
Conversion (take-down and repair) or transtendinous/*in situ* repair without biologic device/implant augmentation	47	56.0%
Conversion (take-down and repair) or transtendinous/*in situ* repair with biologic device/implant augmentation	13	15.5%
Conversion (take-down and repair) or transtendinous/*in situ* repair with PRP	2	2.4%
Conversion (take-down and repair) or transtendinous/*in situ* repair with BMAC	1	1.2%
Other	4	4.8%
Which, if any, of the following surgical augmentation strategies do you currently use in isolation or in addition to a standard repair of partial-thickness rotator cuff tears? (please select all that apply)		
Platelet rich plasma	27	26.7%
Bioinductive collagen implant (eg, REGENETEN)	53	52.5%
In-space balloon	14	13.9%
Bone marrow aspirate concentrate (BMAC)	12	11.9%
Synthetic material patch (nontissue)	11	10.9%
Human allograft augmentation	21	20.8%
None	25	24.8%
Other	5	5.0%

**Table III:** Considerations impacting surgeon decision for use of augmentation for PTT.

Response	# of responses	Mean (CI)
In the absence of augmentation or biologics, on a scale of 1 to 10, with 1 being not challenging and 10 being most challenging, what are the most challenging patient characteristics or treatment aspects when surgically treating partial-thickness rotator cuff tears?		
Poor tendon quality	90	6.5 (5.9-7.1)
Age (ie, > 65 yr old)	91	5.2 (4.7-5.8)
Comorbidities (ie, obesity, smoking, diabetes)	91	7.0 (6.5-7.5)
High grade tears (ie, >50%)	91	5.5 (4.9-6.0)
Preservation of the native footprint	91	4.9 (4.3-5.4)
Prolonged recovery	91	5.3 (4.8-5.8)
On a scale of 1 to 10, with 1 being no impact and 10 being very impactful, what are the most impactful patient selection criteria to your decision to use biologic devices/implants for the surgical treatment partial-thickness rotator cuff tears?		
Patient age <40 yr	82	3.5 (2.9-4.1)
Patient age between 41 and 60 yr	83	4.2 (3.6-4.8)
Patient age >60 yr	84	5.0 (4.3-5.6)
Presence of patient comorbidities (ie, diabetes, current smoker)	84	6.0 (5.4-6.7)
High pre-operative activity level (ie, sport, manual labor)	84	5.4 (4.8-6.1)
High grade (>50%) rotator cuff tear	84	5.4 (4.7-6.1)
Rotator cuff cable is visibly intact	84	4.7 (4.0-5.3)
Full-thickness tear or conversion to full thickness is present	84	5.6 (4.9-6.2)
On a scale of 1 to 10, with 1 being not critical and 10 being very critical, what key outcomes are most critical to your decision to use biologic devices/implants for the surgical treatment of partial-thickness rotator cuff tears?		
Shorter immobilization	82	4.0 (3.3-4.6)
Lower pain in the early post-operative period	82	4.3 (3.7-4.9)
Faster return to activities of daily living	82	5.0 (4.4-5.7)
Faster return to work	82	5.2 (4.5-5.8)
Lower incidence of retear and/or revisions procedures	82	7.0 (6.3-7.7)
Unknown, or no post-operative clinical benefit	81	5.5 (4.7-6.2)

*CI*, confidence interval.

### FTT

Surgical management of FTT preferences included tendon repair without augmentation for 54.8% of surgeons and with augmentation when indicated by 36.9% of surgeons (Table IV). Tendon repair with augmentation was indicated as standard of practice (ie, for all cases of repair) for 7.1% of surgeons. When treating FTT, surgeons identified massive, nonmobilizing retracted tendons (mean: 9.1; 95% CI: 8.7-9.5) and patient comorbidities (mean 7.7; 95% CI: 7.3-8.2) as presenting the greatest challenges to success (Table V). Rotator cuff tear size or number of involved tendons was indicated as impactful when determining whether to use augmentation (mean: 7.6; 95% CI 7.0-8.1) as part of the surgical management. Post-operatively, lower incidence of retear and/or revision procedures was the most critical outcome (mean 8.0; 95% CI: 7.4-8.6) surgeons sought in their decision to use augmentation.

**Table IV:** Management of FTT.

Response	# of responses	Percentage
What is your preferred method of surgical treatment for full-thickness rotator cuff tears?		
Tendon repair without biologic device/implant augmentation	46	54.8%
Tendon repair with biologic device/implant augmentation as standard practice	6	7.1%
Tendon repair with biologic device/implant augmentation only when indicated (ie, specific patient characteristics)	31	36.9%
Other	1	1.2%
Which, if any, of the following surgical augmentation strategies do you currently use in isolation or in addition to a standard repair of full-thickness rotator cuff tears? (please select all that apply)		
Platelet rich plasma	19	18.8%
Bioinductive collagen implant (eg, REGENETEN)	46	45.5%
In-space balloon	30	29.7%
Bone marrow aspirate concentrate (BMAC)	15	14.9%
Synthetic material patch (nontissue)	17	16.8%
Human allograft augmentation	46	45.5%
None	15	14.9%
Other	6	5.9%

**Table V:** Considerations impacting surgeon decision for use of augmentation for FTT.

Response	# of responses	Mean (CI)
In the absence of augmentation or biologics, on a scale of 1 to 10, with 1 being not challenging and 10 being most challenging, what are the most challenging patient characteristics or treatment aspects when surgically treating full-thickness rotator cuff tears?		
Poor tendon quality	91	8.6 (8.2-8.9)
Comorbidities (ie, obesity, smoking, diabetes)	91	6.7 (6.2-7.1)
Age (ie, >65 yr old)	91	7.7 (7.3-8.2)
Small tears (<1 cm)	91	2.9 (2.5-3.3)
Medium tears (1-3 cm)	91	4.2 (3.8-4.7)
Large tears (3-5 cm)	91	6.6 (6.2-7.1)
Massive tears (>5 cm) with mobilizing retraction	91	8.2 (7.9-8.6)
Massive tear (>5 cm) with nonmobilizing retraction	91	9.1 (8.7-9.5)
On a scale of 1 to 10, with 1 being no impact and 10 being very impactful, what are the most impactful patient selection criteria to your decision to use biologic devices/implants for the surgical treatment of full-thickness rotator cuff tears?		
Patient age <40 yr	81	3.5 (2.9-4.2)
Patient age between 41 and 60 yr	82	4.4 (3.8-5.0)
Patient age >60 yr	82	5.6 (5.0-6.3)
Presence of patient comorbidities (ie, diabetes, current smoker)	83	6.5 (5.9-7.2)
Rotator cuff cable is visibly not intact	82	5.6 (5.0-6.2)
Rotator cuff tear size or number of torn tendons present	83	7.6 (7.0-8.1)
Inability to re-establish the native footprint	82	7.2 (6.6-7.8)
On a scale of 1 to 10, with 1 being not critical and 10 being very critical, what key outcomes are most critical to your decision to use biologic devices/implants for the surgical treatment of full-thickness rotator cuff tears?		
Shorter immobilization	82	3.4 (2.8-3.9)
Lower pain in the early post-operative period	82	3.9 (3.3-4.5)
Faster return to activities of daily living	82	4.3 (3.7-4.9)
Faster return to work	82	4.6 (4.0-5.2)
Lower incidence of retear and/or revisions procedures	83	8.0 (7.4-8.6)

*CI*, confidence interval.

### Evaluation of augmentation options

Surgeons indicated that the availability of evidence or research demonstrating positive clinical outcomes was most heavily weighted in their product selection decision for rotator cuff repair (RCR) augmentation (mean: 9.4; 95% CI: 9.2-9.6) (Table VI). Having a clearly defined patient population target for the product was also strongly weighted as a deciding factor (mean: 8.4; 95% CI: 8.0-8.8).

**Table VI:** Critical product information.

Response	# of responses	Mean (CI)
On a scale of 1 to 10, with 1 being not critical and 10 being very critical, what information would be critical to your decision to use biologics devices/implants for the surgical treatment of rotator cuff tears?		
Patient advocating for advanced technology, such as biologic devices/implants	90	5.3 (4.7-5.9)
Research defined target patient population (ie, risk factors, comorbidities)	91	8.4 (8.0-8.8)
Research demonstrating positive clinical outcomes	91	9.4 (9.2-9.6)
Published cost effectiveness and/or utility	90	8.0 (7.6-8.4)

*CI*, confidence interval.

## Discussion

This survey by the ASES Bio-Advocacy Work Group was conducted to quantify real-world clinical practice uses and access patterns for augmentation of rotator cuff tear repair, with a focus on biologic technology options. The findings confirmed our hypothesis that the use of augmentation is popular among shoulder and elbow experts, but that there are limitations in access to the technology for their patients. Based on the survey responses, the Work Group summarized the survey results in 4 statements:

1) RCR augmentation is utilized by a significant majority of surgeons for both PTT and FTT. However, three-fourths of surgeons indicate that they suffer limited or variable access to biologic augmentation options.
2) BCI is the most preferred form of augmentation for PTT, while the BCI and HDA are most preferred for FTT.
3) The evidence-based decision to use a specific augmentation option is largely based on evidence of positive clinical outcomes and a defined target patient population, with the most critical outcome that augmentation can improve reported as lower retear rate for both PTT and FTT.
4) For PTT, patient comorbidities are of greatest concern and are the most impactful criteria for selecting to use biologic devices/implants. For FTT, poor tendon quality and increasing tear size are of greatest concern, with tear size indicated as the most impactful criteria for selecting to use biologic devices/implants.

## PTT

Consistent with previous literature and current AAOS CPGs,^1^^,^^16^ almost 9 out of 10 survey respondents reported preference for surgical intervention for symptomatic PTT of the rotator cuff, indicting a strong consensus in clinical practice. Over half of the survey respondents indicated that a sutured repair, via transtendinous technique or conversion to full-thickness with repair, was their preferred technique. A recent systematic literature review by Longo et al^27^ concluded that these 2 techniques are most commonly used for PTT involving >50% of the tendon and are preferred due to the reduction of risk of tear progression. While patient-reported outcomes are generally good after surgery, transtendinous repairs have a greater propensity for developing post-operative stiffness, while conversion to full-thickness procedures risks altering the native rotator cuff footprint.

Survey respondents rated patient comorbidities as the most challenging factor when treating PTTs, and this was the most impactful patient selection criteria for the use of augmentation. Systemic comorbidities, such as diabetes and hypercholesterolemia or hyperlipidemia, have been reported in 37% and 69% (respectively) of patients with PTT, and may cause poor tissue quality and decrease rotator cuff healing potential post-operatively.^3^^,^^33^ While studies have shown that FTT are more likely to retear than partial-thickness tears after repair,^23^ retear risk was identified by survey respondents as the most critical outcome when considering whether to use augmentation to address a partial thickness tear. Several augmentation options have been shown in the literature to help improve retear risk in cases of PTT.^16^^,^^28^^,^^39^^,^^41^^,^^44^

## FTT

FTT are most commonly treated with a sutured repair to restore the mechanical structure of the torn tendon. Uncertainty remains regarding the best surgical technique, with single- and double-row repairs both reported to provide good clinical outcome and similar post-operative retear rates in smaller tears, but with some evidence that double-row repair may be superior and more cost-effective in larger tears.^1^^,^^19^^,^^25^ Repair technique was not collected as part of this survey, but 54.8% of survey participants indicated that a standard repair without augmentation was their preferred standard of care. The cumulative incidence of post-operative retears following FTT repair across all tear types and repair techniques has been reported around 20%-25%, with increasing rates of failure as tear size increases.^20^^,^^29^ Survey responses reflected this same trend, with surgeons reporting an increasing average rating of challenging tear characteristics from small (2.9) to nonmobile massive tears (9.1).

Due to the multifactorial patient-specific and tear-specific factors identified as challenges for the treatment of FTT, it was no surprise that 85% of respondents in this survey rated retear rate as the most critical outcome when deciding to use augmentation. The most commonly preferred augmentation implants included BCI (46%) and HDA (46%). While this survey did not further explore indications for each augmentation method, the 2 primary augmentation types align with the risk factors and challenges indicated by respondents, as both options have been shown to help with this problem.^1^^,^^13^^,^^28^^,^^43^^,^^44^ Tear size was identified as the most impactful patient factor leading to use of augmentation, and nonmobile, massive tears were reported to be the most challenging aspect of rotator cuff repair. The literature contains multiple reports on the use of HDA for large/massive tears, in which the allograft provides strength and bridging possibilities for mechanical stabilization.^28^^,^^41^^,^^42^^,^^44^ In contrast, the use of BCI has been reported in literature as a supplement when the native footprint can be restored without tension.^7^, ^8^, ^9^, ^10^^,^^30^^,^^31^^,^^37^ Beyond tear size, poor tendon quality (8.6) and advancing age (7.7) were also indicated as challenges impacting decisions surrounding RCR augmentation.

## Limited technology access

The most concerning finding of this study is the limited access to augmentation technology for surgeons and their patients. RCR augmentation is utilized by over three-fourths of survey responders, with 76.2% using augmentation for PTT and 85.1% for FTT. A previous survey of 230 members from ASES and the American Orthopedic Society for Sports Medicine (AOSSM) by Vaudreuil et al^42^ reported similar utilization rates, with 61% of surgeons using human allograft or collagen implants to augment repairs of massive rotator cuff tears. Recently published AAOS CPGs give a “strong” recommendation (4/4 stars) in favor of using BCI and a “moderate” recommendation (3/4 stars) in favor of using HDA for reduction of retear rates and improvement of patient outcomes.^1^ In spite of these documented surgeon preferences, growing clinical evidence, and AAOS recommendations supporting biologic augmentation, only 25% of surgeons in this study reported unrestricted access to augmentation options.

These restrictions are likely directly related to reimbursement factors, as many payors refuse or limit coverage of various implants—often based on site of service. Indeed, 38.9% of surgeons in this survey reported variable access to augmentation technology based on setting of care. Previous studies have also demonstrated similar access problems for patients needing shoulder care. Jarrett et al^21^ documented increased waiting times and site-of-care differentials for shoulder arthroscopy patients with governmental versus private insurance. Donovan et al^15^ exposed declining reimbursements for ASC-based shoulder arthroscopy procedures over the past decade, in spite of a dominant patient and physician preference for ASC's as the site of service. Kassam et al and Saunders et al have likewise shown the effects of payor reimbursement on access to care.^22^^,^^38^ Reiad et al^35^ have shown that some of these disparities in access may play out along racial and ethnic lines. Many payors continue to consider augmentation as “experimental” and “investigational” despite over a decade of high-quality studies and CPGs supporting otherwise.^1^^,^^4^, ^5^, ^6^, ^7^, ^8^, ^9^, ^10^, ^11^^,^^24^^,^^30^, ^31^, ^32^^,^^34^^,^^36^^,^^37^^,^^39^^,^^40^^,^^46^ In addition, payment rates for surgical RCR procedures are not often adjusted to accommodate the incremental costs of augmentation, particularly in specific settings of care. The lack of access to these preferred and recommended augmentation options thus places surgeons at risk for moral injury—a well-documented situation wherein third-party financial interests outweigh evidence-based best practice, leading to inferior care for the patient as well as stress and burnout for the surgeon.^14^^,^^18^^,^^26^ As such, the Work Group recommends further investigation about and solutions for barriers to access for rotator cuff augmentation technology.

## Limitations

This study did have some important limitations. Like all survey studies, we had the inherent weaknesses of recall bias, social desirability bias, nonresponse bias, and lack of generalizability. Most notably, the response rate for the survey was only 8.5% (103/1,210) in spite of our efforts to maximize participation, and only ASES Members received the survey. As such, these results may not necessarily be generalizable to a broader population of surgeons. Ironically, the low rate of participation in this advocacy-themed survey reflects similarly low rates of participation in advocacy activities by orthopedic shoulder surgeons^2^^,^^12^—a longstanding statistic that we hope to see improve. Although we employed multiple emails, collegial encouragement, financial incentives (gift card raffle for participants), and other techniques, the rate of participation was still relatively small. Secondly, surgeons with no experience with augmentation may have foregone survey participation altogether, further affecting reported percentages of use. Third, grouping all augmentation options together for certain survey questions could potentially influence surgeon's answers, especially if they have limited experience with certain options. Fourth, we did not attempt to analyze actual frequency of utilization by surgeons of each type of augmentation option, as this would significantly increase the data load of the survey and seemed beyond the focus of this study. Future studies, however, need to investigate this important concept of utilization frequency. Finally, the digital survey format did not allow for free-text answers to each question, potentially limiting collection of more nuanced responses. In spite of these limitations, however, the Work Group still feels that the findings of this survey, unique in its design, remain important as a pilot study into this rapidly expanding area of clinical care.

## Conclusion

The results of this expert opinion survey confirmed the growing popularity of augmentation of RCRs as well as the significant barriers to access faced by surgeons and their patients. BCI and HDA are the current most popular augmentation options. Surgeons identify multiple factors as important to decision-making for implant use, including positive clinical outcomes, low retear rates, defined patient populations, patient comorbidities, poor tendon quality, and tear size. Research in this area continues to expand and drive formal CPGs, but additional work on payor approval remains to ensure appropriate access to this technology.

## Supplementary Data

Purpose Statement 1
