# Supplementary material for: Augmentation for rotator cuff repair – clinical use patterns and limited patient access: the American Shoulder and Elbow Surgeons bio-advocacy work group survey
Source: JSES Rev Rep Tech. 2026 Apr 10;6(3):100748. doi: 10.1016/j.xrrt.2026.100748 (PMC13185863; doi:10.1016/j.xrrt.2026.100748)
Supplement: Purpose Statement 1 [file mmc1.docx]

**Purpose Statement #1:** The purpose of this study is to evaluate the use of *surgical augmentation* and the specific use of *biologic devices/implants,* for the treatment of partial- and full-thickness rotator cuff tears.

1. Which, if any, of the following *surgical augmentation* strategies do you currently use either in isolation or in addition to a standard repair of *partial*-thickness rotator cuff tears? (Please select all that apply.)
2. Platelet Rich Plasma
3. Bioinductive collagen implant (e.g., REGENETEN)
4. In-space Balloon
5. Bone marrow aspirate concentrate (BMAC)
6. Synthetic material patch (non-tissue)
7. Human allograft augmentation
8. Which, if any, of the following *surgical augmentation* strategies do you currently use in isolation or in addition to a standard repair of *full*-thickness rotator cuff tears? (Please select all that apply.)
9. Platelet Rich Plasma
10. Bioinductive collagen implant (e.g., REGENETEN)
11. In-space Balloon
12. Bone marrow aspirate concentrate (BMAC)
13. Synthetic material patch (non-tissue)
14. Human allograft augmentation

**Survey Instructions:** The remaining questions are **only** about biologic devices and/or implants that are used in isolation and as an augment during surgical intervention for a rotator cuff tear.

1. Which statement accurately describes the restrictions on your current selection and use of *biologic devices/implants augmentation* for the *surgical* treatment for rotator cuff tears based on insurance payment rates or methodologies (i.e., packaged payments for all implants and supplies)?
2. I have no restrictions or unaware of any restrictions on biologic devices/implants used for the surgical treatment of rotator cuff tears
3. I am limited on the type of biologic devices/implants used for the surgical treatment of rotator cuff tears
4. I have no access to biologic devices/implants used for the surgical treatment of rotator cuff tears
5. My access to biologic devices/implants used for the surgical treatment of rotator cuff tears is variable based on setting of surgical care (i.e. ASC vs Hospital)
6. On a scale of 1 to 10, with 1 being not critical and 10 being very critical, what information would be critical to your decision to use biologics devices/implants for the surgical treatment of rotator cuff tears?
   1. Patient advocating for advanced technology, such as biologic devices/implants
   2. Research defined target patient population (i.e. risk factors, comorbidities)
   3. Research demonstrating positive clinical outcomes
   4. Published cost effectiveness and/or utility
   5. Reflected in Clinical Practice Guidelines
7. In the absence of augmentation or biologics, on a scale of 1 to 10, with 1 being not challenging and 10 being most challenging, what are the most challenging patient characteristics or treatment aspects when surgically treating partial-thickness rotator cuff tears?
   1. Poor tendon quality
   2. Age (i.e. >65 years old)
   3. Comorbidities (i.e. obesity, smoking, diabetes)
   4. High grade tears (i.e. >50%)
   5. Preservation of the native footprint
   6. Prolonged recovery
8. In the absence of augmentation or biologics, on a scale of 1 to 10, with 1 being not challenging and 10 being most challenging, what are the most challenging patient characteristics or treatment aspects when surgically treating full-thickness rotator cuff tears?
   1. Poor tendon quality
   2. Comorbidities (i.e. obesity, smoking, diabetes)
   3. Age (i.e. >65 years old)
   4. Small Tears (<1 cm)
   5. Medium Tears (1-3cm)
   6. Large Tears (3-5cm)
   7. Massive tears (>5cm), with mobilizing retraction
   8. Massive tear (>5cm), with non-mobilizing retraction

**Survey Instructions**: For the following questions, please assume (1) the patient has *failed* 3 or more months of conservative treatment and (2) ***surgical reimbursement is not a barrier.***

1. What is your *preferred* method of surgical treatment for partial-thickness rotator cuff tears that have failed conservative management?
2. This is still not a surgical case, conservative treatment remains the primary treatment option
3. Subacromial decompression and debridement only
4. Biceps treatment (i.e. tenotomy, tenodesis) procedure only
5. No rotator cuff repair and isolated use of biologic device/implants
6. Conversion (take-down and repair) or transtendinous/in-situ repair without biologic device/implant augmentation
7. Conversion (take-down and repair) or transtendinous/in-situ repair with biologic device/implant augmentation
8. Conversion (take-down and repair) or transtendinous/in-situ repair with PRP
9. Conversion (take-down and repair) or transtendinous/in-situ repair with BMAC
10. On a scale of 1 to 10, with 1 being no impact and 10 being very impactful, what are the most impactful patient selection criteria to your decision to use biologic devices/implants for the surgical treatment partial-thickness rotator cuff tears?
11. Patient age <40 years
12. Patient age between 41-60 years
13. Patient age >60 years
14. Presence of patient comorbidities (i.e. diabetes, current smoker)
15. High pre-operative activity level (i.e. sport, manual labor)
16. High grade (>50%) rotator cuff tear
17. Rotator cuff cable is visibly intact
18. Full-thickness tear or conversion to full thickness is present
19. On a scale of 1 to 10, with 1 being not critical and 10 being very critical, what key outcomes are most critical to your decision to use biologic devices/implants for the surgical treatment of partial-thickness rotator cuff tears?
20. Shorter immobilization
21. Lower pain in the early post-operative period
22. Faster return to activities of daily living
23. Faster return to work
24. Lower incidence of retear and/or revisions procedures
25. Unknown, or no post-operative clinical benefit
26. What is your *preferred* method of surgical treatment for full-thickness rotator cuff tears?
    1. Tendon repair *without* biologic device/implant augmentation
    2. Tendon repair, *with* biologic device/implant augmentation as standard practice
    3. Tendon repair, with biologic device/implant augmentation only when indicated (i.e. specific patient characteristics)
27. On a scale of 1 to 10, with 1 being no impact and 10 being very impactful, what are the most impactful patient selection criteria to your decision to use biologic devices/implants for the surgical treatment of full-thickness rotator cuff tears?
    1. Patient age <40 years
    2. Patient age between 41-60 years
    3. Patient age >60 years
    4. Presence of patient comorbidities (i.e. diabetes, current smoker)
    5. Rotator cuff cable is visibly not-intact
    6. Rotator cuff tear size or number of torn tendons present
    7. Inability to re-establish the native footprint
28. On a scale of 1 to 10, with 1 being not critical and 10 being very critical, what key outcomes are most critical to your decision to use biologic devices/implants for the surgical treatment of full-thickness rotator cuff tears?
29. Shorter immobilization
30. Lower pain in the early post-operative period
31. Faster return to activities of daily living
32. Faster return to work
33. Lower incidence of retear and/or revisions procedures
